# Bovine leukemia virus discovered in human blood

**DOI:** 10.1186/s12879-019-3891-9

**Published:** 2019-04-02

**Authors:** Gertrude C. Buehring, Anne DeLaney, HuaMin Shen, David L. Chu, Niema Razavian, Daniel A. Schwartz, Zach R. Demkovich, Michael N. Bates

**Affiliations:** 1 School of Public Health, University of California, 16 Barker Hall, Berkeley, CA 94720-7354 USA; 2 Kaiser Permanente Medical Cente, San Rafael, CA USA; 3 Oakland University William Beaumont School of Medicine, Oakland, MI USA; 4 University of Michigan Medical School, Ann Arbor, MI 48109 USA; 5 Joint Medical Program, University of California, Berkeley, CA USA; 6 University of California, San Francisco, CA USA; 7 RTI International, San Francisco, CA USA

**Keywords:** Bovine leukemia virus, Human blood, Zoonotic infection

## Abstract

**Background:**

Bovine leukemia virus (BLV) infection is widespread in cattle globally and is present in marketed beef and dairy products. Human infection with BLV has been reported in breast and lung cancer tissues and was significantly associated with breast cancer in 3 case-control studies. The purpose of this current research was to determine if BLV is present in human blood cells and if antibodies to BLV are related to blood cell infection.

**Methods:**

Standard liquid PCR and Sanger DNA sequencing were used to test for BLV in buffy coat cells (leukocytes and platelets) of blood specimens from 95 self-selected female subjects.

Enzyme-linked immunosorbent assay (ELISA) for IgG, IgM, and IgA was used to detect antibodies to BLV in the plasma of the corresponding blood samples.

**Results:**

BLV DNA was detected in the buffy coat cells of blood in 33/95 (38%) of the subjects by PCR and DNA sequencing. IgG antibodies were detected in 30/95(32%), IgM in 55/95(58%), and IgA in 30/95(32%) of the subjects. There was no significant correlation between presence of the antibodies and presence of BLV DNA.

**Conclusions:**

This first report of BLV in human blood raises the question of whether infection of leukocytes could conceivably lead to leukemia as it does in infected cattle. Also, system wide circulation of infected blood cells could facilitate BLV transit to various internal tissues/organs with potential for their infection and subsequent development of cancer. The most likely route of BLV transmission to humans would be zoonotic, as a foodborne infection. Although eradicated from cattle in some countries, BLV still has a high rate of infection in the Americas, the Middle East, and parts of Europe and Asia. This report of BLV in the blood layer containing human leukocytes/platelets adds important information which could be useful to elucidate possible routes of transmission of BLV to humans and to prevent further human infection.

## Background

Bovine leukemia virus (BLV) is an oncogenic deltaretrovirus that is emerging as a possible zoonotic infection. BLV is widespread globally in domesticated cattle, especially in the Americas, and parts of Europe, Asia and the Middle East. In the USA, 84% of US dairy herds and 39% of beef herds are infected [1]. Only 5% of infected animals develop leukemia or lymphoma, requiring exclusion of their products from the agricultural market. The remaining 95% of the infected animals remain subclinical with persistent lymphocytosis, and are a major source of beef and dairy products [2, 3]. In cattle BLV is found primarily in blood lymphocytes (B cells), endothelial cells [2], and in mammary epithelial cells (MEC), which frequently exfoliate into milk [4].

BLV infects a few species naturally, especially if they are near cattle: water buffalo, sheep, alpaca [5, 6]. It has been experimentally transmitted to rabbits, rats, pigs, goats, and sheep [5]. Evidence that BLV infects humans has been accumulating over the past 5 years. Although BLV is classified as an RNA virus (deltaretrovirus family), upon entry into a cell, it rapidly makes a DNA copy of its genome with its reverse transcriptase enzyme, and this retrotranscribed DNA is what predominates in infected cells [5]. Retrotranscribed BLV DNA has been independently identified in the breast tissue (both benign and malignant) of human females in Columbia [7], the USA [8, 9], Australia [10], and Argentina [11] in 5 separate investigations using standard liquid PCR and/or in situ PCR. These studies obtained slightly different frequencies of women whose breast tissues were positive for BLV, which would be expected because the proportions of women with breast cancer vs. normal controls were different and the populations were from different countries with distinct variations in ethnicity and dietary preferences: Columbia = 43/105 (41%) [7]; Australia = 59/96 (61%) [10]; Argentina = 12/25(48%) [11]; and two different regions of the USA: East and Southeast = 97/218 (44%) [8], and Texas = 73/214 (34%) [9]. BLV was also detected in 8/10 [80%] human squamous cell lung carcinomas by a sequence based methodology using a microbial detection microarray that detects all viral and bacterial families whose genomes have been sequenced [12]. This microarray method is estimated to have a sensitivity somewhat less than standard PCR but more than next generation sequencing (NGS), which detects only viral DNA integrated into the human genome, often present in concentrations too low (< 1% of the reads) to be detected without amplification [13].

These previous studies identifying BLV in human tissues emphasize the need to determine how BLV infects humans. Based on the predominance of BLV in blood leukocytes of infected cattle and its common transmission to other cattle via blood, the goal of this study was to determine if BLV is present in human blood leukocytes, an essential first step to determine the route of transmission of BLV to humans and how the initial infection might spread to secondary sites.

## Methods

### Study population

The study population was a self-selected convenience population of 95 female patients at Kaiser Permanente Hospital, San Rafael, CA, responding to recruitment flyers posted in patient waiting rooms. Participating volunteers signed informed consent agreements to have an extra tube of blood drawn for the study during the routine pre-operative blood draw the day before scheduled surgeries of various types. Use of human subjects was approved by Kaiser Permanente Northern California Institutional Review Board and the University of California, Berkeley (UCB) Committee for the Protection of Human Subjects.

### Blood processing

Blood specimens were obtained from the clinical laboratory where the blood had been drawn into a tube containing an anti-coagulant and refrigerated at 4 °C. After transfer of specimens to the research laboratory at UCB 2–14 days later, blood was centrifuged (500×g for 10 min). Degree of hemolysis (erythrocyte rupture) in the plasma was subjectively judged as a possible indicator of blood specimen deterioration, and recorded as no hemolysis (normal pale yellow color of plasma), slight hemolysis (slightly pink color), or moderate hemolysis (pale red color). No samples were completely hemolyzed and only 2 out of 95 were moderately hemolyzed. After removal of plasma, the intact visible leukocyte and platelet-rich buffy coat above the red blood cells was transferred to a separate tube. Both plasma and buffy coat specimens were frozen at − 20 °C. until they were used for analysis.

### DNA extraction

DNA was extracted from the blood buffy coat layer using the QIAamp® DNA mini kit (Qiagen, Valencia, CA, USA, catalogue #51304) according to manufacturer’s instruction. The positive control cell line was FLK, a sheep cell line derived from fetal lamb kidney and infected with BLV [14]**.** FLK monolayers were detached from their substrate by standard saline-trypsin-versene (STV) solution, rinsed with Dulbecco’s modified phosphate buffered saline (DPBS) and pelleted (500×g) before DNA extraction. The negative control for PCR was sterile, filtered distilled water added to the reaction mix in the same volume as the samples added to the reaction mix**.** The quality of extracted DNA from each human blood specimen was confirmed by electrophoreses (100 V for 30 min) on a 1.5% agarose gel in Tris, boric acid, EDTA (TBE) buffer to detect an ethidium bromide stained amplified segment of the gene for the human housekeeping enzyme, glyceraldehyde-3-phosphate dehydrogenase (GAPDH). A strong compact band at the correct position on the gel indicated high quality DNA suitable and in sufficient amount for PCR and sequencing.

### PCR

For amplification of BLV that might be present in the sample, Taq polymerase (Promega GoTaq® Flexi DNA Polymerase, catalogue #M8296) was used in a standard liquid PCR procedure. PCR primers and cycling conditions for GAPDH are as follows:

3′-5′ GAGTCAACGGATTTGGTCGT

5′-3′ TTGATTTTGGAGGGATCTCG

using cycling condition as follows: 36 cycles: 95 °C – 2 min; 95 °C – 30 s, 50 °C – 30 s, 72 °C – 22 s; 1 cycle 72 °C – 5 min.

All specimens positive for BLV were checked for a sheep housekeeping gene to rule out contamination from the positive control cell line, derived from a lamb’s kidney. PCR primers and cycling conditions for sheep cytochrome C oxidase are as follows [15]:

3′-5′ CGATACACGGGCTTACTTCACG

5′-3′ AAATACAGCTCCTATTGATAAT

using cycling condition as follows: 35 cycles: 95 °C – 2 min; 95 °C – 30 s, 53 °C – 30 s, 72 °C – 24 s; 1 cycle 72 °C – 5 min.

Standard liquid PCR using nested primers was used to detect BLV in DNA extracted from buffy coat cells. The BLV primers used were from the long terminal repeat (LTR) promoter region, the *gag* region coding for the p24 capsid protein, the *env* region coding for the gp51 envelope protein, and the *tax* region coding for the oncogenic protein. Each genome region was tested individually because the individual pairs of primers required different reaction conditions. Table 1 presents the primer specifics.

**Table 1:** Primers and reaction conditions used to detect BLV DNA in human buffy coat cells

BLV gene	Primer sequences 5′ to 3′	Location in bp*	Nested PCR role	Product length, bp	Annealing temp, °C.	Extension time, s
LTR	F: TAGGAGCCGCCACCGC	23–38	Outer	329	57	22
	R: GCGGTGGTCTCAGCCGA	352–336				
	F: CGTAAACCAGACAGAGACG	41–59	Inner	290	58	20
	R: CACCCTCCAAACCGTGCTTG	331–312				
*gag*	F: AACACTACGACTTGCAATCC	1068–1087	Outer	385	54	28
	R:GGTTCCTTAGGACTCCGTCG	1453–1434				
	F: ACCCTACTCCGGCTGACCTA	1097–1116	Inner	272	56	24
	R:CTTGGACGATGGTGGACCAA	1369–1350				
*env*	F:CGGGCAAAACAATCGTCGGT	4701–4720	Outer	707	55	45
	R:ACTGGGTTCCCTCTGTCAGA	5408–5389				
	F: CTCTCCTGGCTACTGACC	4763–4780	Inner	611	55	45
	R: GGAAAGTCGGGTTGAGGG	5374–5357				
*tax*	F: TATTCCACCTCGGCAC	7153–7169	Outer	447	50	28
	R: ATTGGCATTGGTAGGGCT	7600–7583				
	F: CTTCGGGATCCATTACCTGA	7197–7216	Inner	373	55	24
	R: GCTCGAAGGGGGAAAGTGAA	7570–7551				

Abbreviations: *bp* base pair, *F* forward, *R* reverse, s seconds, *temp* temperature; bp* Base pair numbering is according to GenBank reference sequence EF600069

Reverse primer sequences for GAPDH and all BLV genome regions are presented in reversed order and complementary to the proviral reference sequence

### DNA sequencing

Amplified DNA sequences were purified using Zymoclean™ Gel DNA Recovery Kit (Zymo Research, Irvine, CA, Catalogue # D4007) before sending a 25-100 ng sample (depending on sequence length) to the UCB DNA Sequencing Facility for Sanger sequencing. Sequences were run in both forward and verse directions and checked against corresponding electropherograms. They were accepted as readable only if they matched in both directions and each base was clearly identifiable, i.e. no “N’s” were indicated in the sequences.

### Precautions to prevent DNA cross contamination

Throughout all laboratory work with initial specimens and DNA, special precautions were used to prevent cross contamination among individual specimens and the positive control: separate rooms/work units dedicated to a particular step of the procedure, e.g. DNA-free room to prepare PCR reaction mix; special hood with UV light and nucleic acid decontamination solutions (RNAse AWAY, Molecular Bioproducts, San Diego, CA, USA) for the addition of DNA to the reaction mix; dedicated biohazard hood for all work with the positive control cell line, and fume hood with external exhaust for all work with the positive control DNA.

### Enzyme-linked immunosorbent assay (ELISA) for anti-BLV antibody detection

Indirect ELISA was used to assess three isotypes (IgG, IgM, and IgA) of serum antibodies to BLV p24 capsid protein. ELISA plates (Immulon 2HB, Thermo Fisher Scientific, Waltham, MA) were coated with 1250 ng/well recombinant BLV capsid p24 antigen (formerly sold by Synbiotics, San Diego, CA)*.* Antigen concentration was 1250 ng/well, diluted in 200 μl carbonate-bicarbonate coating buffer (15 mM Na_2_CO_3_, 35 mM NaHCO3, pH 9.6) plus 0.0002% purified BSA (bovine serum albumin). After overnight incubation at 4 °C, coating buffer was removed and wells were washed for 5 min with ELISA wash buffer (DPBS with 0.055 Tween 20). Wells were then incubated 1 h at room temperature with 1.5% bovine serum albumin (BSA) in DPBS to block non-specific reactions. Plates were washed with wash buffer for 5 min after each subsequent step, except blocking and detection steps. All reactions and wash steps utilized a 200 μl volume and were performed at room temperature. Primary antibody was the human blood plasma specimen diluted 1:100 in wash buffer and reacted 120 min. Secondary antibody was a biotinylated goat anti-human antibody specific for IgG, IgM, or IgA (Vector Laboratories Burlingame, CA) diluted 1:67 in wash buffer and reacted for 120 min. The biotin marker on the adhering secondary antibody was detected using VECTASTAIN ABC reagent (Vector Laboratories) and the chromagen, 3,3′-diaminobenzidine (Sigma Aldrich, St. Louis, MO), reconstituted according to manufacturer’s instructions, and reacted with test samples for 10 min. After removal of the chromagen, 100 μl distilled water was added to each well. Optical density was measured at 492 nm in a SpectraMax M2 ELISA reader (Molecular Devices, Sunnyvale, CA). The plate was blanked on a well containing only distilled water. All samples were run in triplicate. During each assay, the following controls were run to insure accuracy: one known positive and one known negative for each antibody isotype, as determined in a previous study by immunoblotting [16], the gold standard test for antibody detection [17]. In addition, a secondary antibody control, using wash buffer in place of primary antibody, was used to adjust for any non-specific binding of the secondary antibody.

Samples were classed as positive or negative based on cutoff values determined by ROC.

(receiver operating characteristic) curves [18]. The range of sensitivity and specificity values plotted on the ROC y and x axes respectively, were based on samples determined to be positive and negative in a previous study using immunoblotting, more specific for detecting anti-BLV antibodies in cattle serum [16]. ROC modifications correct for potentially false positive ELISA values, reducing the number of positive samples, but increasing the specificity of the assay.

### Statistical analysis

Specimens were considered positive or negative for each of the primary genome regions tested (LTR, *tax, gag)* only if positive PCR results were obtained at least twice, each in independent PCR assay batches. Raw data were uploaded onto STATA 14 for analysis [19]. Prevalence of BLV in blood was computed using base functions. Association of BLV presence with donor age, degree of blood sample hemolysis, and presence of antibody isotypes were each determined using unconditional multivariable logistic regression [19]. Using standard statistical procedures, *P* values were derived from Pearson chi square tests, or Mann-Whitney U test.

## Results

**Fig. 1:**
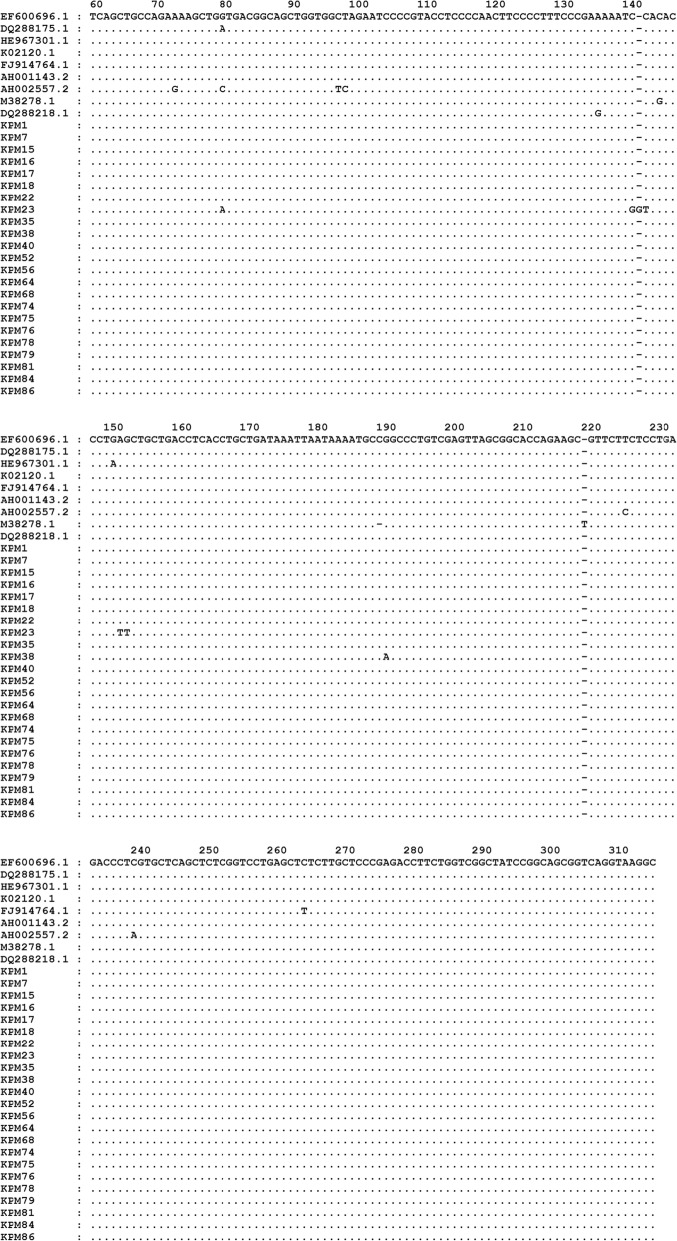
Partial sequences of the long terminal repeat (LTR) promoter region of BLV based on DNA from blood cells of the 23 KPM study subjects positive for the LTR (long terminal repeat) promoter region of BLV. These sequences are compared to 9 GenBank reference sequences [20] (top left column) from 6 of the 10 BLV genotypes established through comparisons of the *env* sequences of BLV isolated from cattle [1]. No reference sequences of the LTR region were available in GenBank from 3 of the genotype groups (7, 10,11). The reference sequence accession code, specimen country of origin, and genotype group are as follows (genotypes in parentheses are probable, based on country of origin, but not proven by phylogenetics): EF600696.1 and DQ288175.1 - USA, (genotype 1 or 3); HE967301.1 – Uruguay, (genotype 1); K02120.1 – Japan, (genotype 1 or 3); FJ914764.1 – Argentina, genotype 2; AH001143.2 and AH002557.2 – Belgium, (genotype 4); M38278.1 – Russia, (genotype 4,7, or 8); DQ288218 – Costa Rica, genotype 5; The first base of each 10 bases is directly under the first digit of the base pair (bp) number. Dots indicate nucleotide bases identical to the consensus. Letters indicate bases differing from the consensus of the reference sequences. Figure formatting was done with GeneDoc (https://genedoc.software.informer.com)

The number of study subjects positive for at least one of three BLV genome regions tested was 36/95 (38%). Frequencies varied for the three genome regions tested: LTR = 22/95(23%), *tax* = 21/95 (22%), *gag* = 12/95(13%), both *tax* and LTR = 12/95 (13%), LTR, *gag, tax =* 5/95 *=* 5%. Comparing BLV positive versus BLV negative samples, there was no significant difference in study subject age (*P* = 0.93; two-tailed Mann-Whitney U test) or degree of blood specimen hemolysis (*P* = 0.828; Pearson chi square test). Only samples positive for the LTR promoter region were sequenced because the LTR is a highly conserved BLV genome region, i.e. not deleted out of the virus genome, and the largest number of subjects were positive for this region. LTR also has the greatest degree of sequence variation among the two highly conserved regions (LTR and *tax*) [20]. The sequences were compared against BLV LTR sequences deposited in Gen Bank [21]. At least one country in each of the 10 BLV genotype groups based on the env region [1] was represented by at least one reference sequence from the LTR region. All readable LTR sequences are presented in Fig. 1. Sequences from 20/22[91%] subjects were an exact match with the consensus sequence of the GenBank reference sequences, including EF600696, derived from the DNA of a US cow [21], and therefore likely to represent the BLV strain that a human population in the USA might be exposed to. Of the two LTR DNA sequences that did not match reference sequences, one (KPM23), had a single base variation at base #80 (G replaced by A) like reference sequence DQ288175, based on DNA isolated from a US cow from the state of Pennsylvania. However, two other variations of KPM23 at bp142–3 and bp152–3 did not match sequence DQ288175 or any other reference sequence. The other human sequence (KPM38) had one variation at base #191 (A replaced by G) which did not match any of the reference sequences deposited in GenBank. The sequence of the FLK positive control cell line is identical to reference sequence EF600696, which was based on the FLK cell line first sequenced in 1985 by other investigators [22].

Two approaches were taken to confirm that BLV LTR sequences matching the positive control cell line were not the result of cross-contamination with DNA from the control cell line. All BLV-positive human DNA samples were assayed for the presence of sheep cytochrome C oxidase, a housekeeping gene unique to sheep [15], the species from which our positive control cell line originated. For samples positive for the *env* region, additional sequencing was performed on the BLV *env* region containing a signature mutation at bp 5194 (G substituted for C), unique to our stock of the FLK cell line and unlike any BLV sequence deposited in GenBank [21]. None of the human BLV-positive samples exhibited either of these markers of positive control cell line contamination. Also, no variations from the reference sequences were seen in the human envelope region.

### Presence of antibodies to the BLV (p24 capsid protein)

Frequency of subjects positive for the three antibody isotypes assayed was as follows: IgG = 30/95(32%), IgM = 55/95 (58%), IgA = 30/95(32%). The difference in frequency of antibody isotypes in BLV-positive versus BLV-negative subjects was not statistically significant. There was also no significant relationship of BLV DNA presence in the blood specimens to any of the three antibody isotypes tested in this study after adjustment for hemolysis and donor age (Tables 2, 3, and 4, *P* values all much greater than 0.05).

**Table 2:** Association of BLV presence in blood with odds of having an IgG antibody to BLV

				Unadjusted		Adjusted	
	All Subjects	IgG+	IgG-	OR (95% CI)	P^1^	OR (95% CI)	P^1^
BLV+	36 (38%)	13 (14%)	23 (24%)	1.40 (0.52–3.69)	0.46	1.40 (0.58–3.38)	0.46
BLV-	59 (62%)	17 (18%)	42 (44%)

**Table 3:** Association of BLV presence in blood with odds of having an IgM antibody to BLV

				Unadjusted		Adjusted	
	All Subjects	IgM+	IgM-	OR (95% CI)	P	OR (95% CI)	P
BLV+	36 (38%)	21 (22%)	15 (16%)	1.03 (0.41–2.61)	0.95	1.03 (0.44–2.38)	0.95
BLV-	59 (62%)	34 (36%)	25 (26%)

**Table 4:** Association of BLV presence in blood with odds of having an IgA antibody to BLV

				Unadjusted		Adjusted	
	All Subjects	IgA+	IgA-	OR (95% CI)	P	OR (95% CI)	P
BLV+	36 (38%)	14 (15%)	22 (23%)	1.71 (0.64–4.52)	0.23	1.71 (0.71–4.13)	0.23
BLV-	59 (62%)	16 (17%)	43 (45%)				

^1^Unadjusted -P values are derived from one-sided chi-squared distribution. Adjusted p-values are derived from two-sided z-test from unconditional logistic regression, adjusting for P age as confounding variable

## Discussion

The results of this study indicate the presence of retrotranscribed bovine leukemia virus (BLV) DNA in blood cells of 36/95(38%) subjects in a self-selected study population. This corroborates previous reports of BLV infection of human breast and lung, and suggests that leukocytes and/or platelets are additional human cell types that can become infected with BLV. It also raises the possibility that BLV infection could initiate additional type(s) of human cancer. Cells infected with BLV and its close relative human T-cell leukemia virus (HTLV) rarely produce infectious extracellular BLV particles and it appears that cell to cell contact is important for viral transmission from an infected to an uninfected cell [5]. Exosomes shed by BLV infected cells of cattle might also facilitate cell to cell transmission of BLV among cattle [23]. Therefore, circulating BLV-infected leukocytes might be efficient agents for virus delivery to diverse tissue types, especially internal organs. In cattle, BLV causes the majority of leukemias and lymphosarcomas [2]. Although non-lymphatic internal organs have not been observed as sites of BLV associated cancer in cattle, this may be because cattle are usually slaughtered at 2–8 years old in a potential lifespan of 20–25 years. This short life may limit the development of many types of cancer and the opportunity to investigate whether BLV might cause cancers of internal organs in older cattle.

Although BLV is classified as an RNA virus (deltaretrovirus family), upon entry into a cell it rapidly makes a DNA copy of its genome with its reverse transcriptase enzyme, a defining characteristic of all retroviruses. The life cycle of BLV does not include host cell assisted production of RNA copies of itself as complete virions for export into the extracellular environment [23]. Also, unlike retroviruses in other families, e.g. HIV, deltaretroviruses do not have their own independent means of passing through the nuclear membrane in order to integrate into the host genome. They can enter the nucleus only during cell division when the nuclear membrane temporarily dissolves [23]. The plentiful linear and circular retrotranscribed DNA genome copies of BLV found within host cell cytoplasm are believed to be copies that never entered the nucleus because host cells were not dividing [23], as opposed to excised copies of BLV previously integrated into host cell DNA and then leaving to enter the cytoplasm [24]. Frequent division of leukocytes does not usually occur in adult humans unless they have untreated leukemia. For these reasons, we targeted BLV DNA rather than RNA as our indicator of BLV presence within human leukocytes, and used standard PCR to amplify this BLV genomic DNA, which is abundant in the cytoplasm of infected cells. Also, our primary goal was to search for current and historic markers of BLV presence (DNA) in human subjects, rather than its current activity (transcribed RNA).

An important strength of the study was the choice of more than one BLV genome region as the target for BLV detection. The LTR (long terminal repeat) promoter region of BLV, and *tax,* coding for the oncogenic protein, were chosen because they are the most highly conserved regions of BLV [20, 25], i.e. the least likely to be deleted from the BLV genome. The *gag* and *env* regions coding for the capsid and envelope proteins, respectively, are the targets for the immune response in cattle and, in BLV and its close relative HTLV (human T-cell leukemia virus), the *gag- pol* (polymerase)- env segment of the BLV genome is often deleted during the progression of leukemias and lymphomas to advanced stages [5, 26], presumably to escape the host’s immune response. Viral detection could possibly be missed if these regions were the primary or sole target for assays. Therefore, although we tested for the *gag* region, we did not include the *env* region for the initial screening for BLV detection, as in cattle, it is commonly deleted [26]. For sequencing, the LTR region was chosen because it shows greater sequence variation (single base substitutions) than *tax* [20, 25]. Base substitutions are valuable for genome comparisons in viruses such as the deltaretroviruses, e.g. BLV and HTLV, which have high genomic stability and a low overall mutation rate compared to other oncogenic retroviral families and to lentiviruses, e.g. HIV [5, 23].

Sequence variations also aid in identifying each specimen and checking for contamination from the positive control cell line and cross contamination among different virus isolates. Neither of the two specimens we obtained with variations from the reference sequences shared the same base substitution or had a base substitution identical to the FLK positive control cell line, as illustrated in Fig. 1, suggesting no cross contamination among DNA specimens from different study participants. Although the overall number of sequence differences among the 7625 nucleotides constituting the 23 specimens sequenced is small and within the range of sequence error for Taq polymerase [27], it is unlikely that they represent Taq polymerase error since the FLK positive control cell line, when sequenced in our laboratory, showed no variation from the Standard Nucleotide BLAST site BLV reference sequence EF600696 which was based on the FLK cell line sequenced in 1985 [22].

Another strength of this study is that the primer sequences were chosen because of their high homology with BLV (E ≤ .28–.31), and low homology with other retroviruses and the human genome including endogenous retroviruses (E = 2.3–750), based on the Standard Nucleotide BLAST option [21]. The purpose of testing primer specificity on the BLAST (Basic Local Alignment Search Tool) site was to compare primer specificity for only two species: bovine leukemia virus and *Homo sapiens*, and to insure that the primers we used were detecting only bovine leukemia virus and not other retroviruses or the human genome, including endogenous retroviruses. Since the BLAST nucleotide data base includes sequences for 49,985,097 different species of organisms, we followed the advice of the BLAST instruction site and narrowed the search in the nucleotide database to bovine leukemia virus only (in box entitled “organism”). We also set the parameters to search only for “highly similar sequences,” which greatly reduces the number of nonspecific matches. When the search results came up we looked only at genomes, not “transcripts” or “protein” matches, since our study was based strictly on DNA genome similarities. E values are a measure of the similarity of two sequences being compared. E values ≤1.00 indicate low probability of random chance similarity and therefore a high specificity as a primer match for the targeted BLV sequence; E values > 1.00 indicate a high probability that sequences being compared are similar due to random chance rather than true relatedness and therefore, as applied to the BLV primers we used, a very low probability that our primers were amplifying human genome sequences including endogenous retroviruses. The strongest confirmation of the specificity of our primers comes from previous laboratory testing which indicated that both the *tax* and LTR primers used here amplified a BLV product, but failed to amplify a product when tested on HTLV (human T-cell leukemia virus) and representatives of all other retroviral and lentiviral families, human papillomavirus, Epstein-Barr virus, and human endogenous retrovirus K [28].

Statistical strengths of the study are that the BLV-positive and BLV-negative subjects were by chance, quite similar in terms of age distribution, reducing the potential for age-related confounding. Also, as internal validations, statistical analyses were performed independently by two persons (M.B. and D.S.), and ELISA assays were performed independently at different times by two persons (N.R. and D.C) in each case blinded as to each other’s results. The two sets of ELISA values were consistent with each other and the final conclusions of the two independently conducted statistical analyses were identical.

Because the BLV DNA was assayed using extracted DNA rather than an in situ technique, we could not verify that the virus was intracellular. However, the source of the DNA was the concentrated buffy coat (leukocytes and platelets) and the strong gel electrophoresis band obtained for the human housekeeping gene GAPDH suggests that cellular DNA was abundant in the DNA extract. The amount of material obtained from buffy coats of 7-8 ml. blood samples, however, was not sufficient to separate leukocytes into different categories and determine which individual cell types were infected, or to investigate protein biomarkers of virion production. These would be important objectives for future studies using blood samples with greater volume. Finally, because clinical information on the self-selected donor population consisted only of age and gender, it was impossible to investigate the association of BLV presence in leukocytes with any specific diseases.

Results of the antibody ELISA assays were consistent with those of a previous study using immunoblotting [16]. Both studies showed that humans have IgG, IgM, and IgA antibodies to BLV. Although detection of antibodies to viruses is a common and extremely useful means of diagnosing viral diseases, in the case of BLV infections, relying on antibodies to prove infection has several disadvantages. BLV may not express p24 capsid protein in blood cells and may not replicate there. Studies in cattle indicate that lymphocytes harboring BLV provirus rarely produce extracellular virions or express viral proteins even though the cattle have antibodies to BLV [16]. The exact site(s) of viral expression in cows that stimulate the production of serum antibodies against BLV was elusive for decades [1]. However, in 1994 when cells isolated from the milk of lactating dairy cows were tested, a high level of p24 was detected within the mammary epithelial cells of 10/28(36%) cows [4]. In humans, a previous study on breast tissue specimens indicated that only 12/215 (6%) of specimens positive for BLV by PCR showed p24 expression in mammary epithelial cells [28]. A possible explanation for the greater frequency of BLVp24 expression in cattle could relate to hormones. Dairy and beef cows are kept in a constant state of pregnancy and lactation during their adult life, whereas most human females are not. BLV genomic transcription is hormone responsive via a hormone response element in the LTR region [29] that is stimulated by progesterone and corticosteroids [30]. In the current and previous studies on BLV in humans, most subjects were beyond the usual age range for pregnancy and lactation, the reproductive phases during which human progesterone and cortisol levels are highest. At human parturition, maternal progesterone is 6x higher and cortisol 70x higher than in the nonpregnant state [31]. In this study, we were unable to determine the association between antibody presence and reproductive phase the subject may have been in at the time blood was drawn. The limited information available on each donor did not include history of pregnancy and lactation, or use of corticosteroid medications.

For antibody isotypes IgG and IgA, BLV-positive subjects were more likely to have anti-BLV antibodies than BLV-negative subjects. However, the differences were not statistically significant. The most probable explanation for the presence of human antibodies to BLV is an immune reaction to heat inactivated BLV consumed in pasteurized dairy products and cooked beef products. Numerous studies in cattle indicated that vaccination of cattle with inactivated, non-infectious BLV resulted in production of antibodies to BLV, although the strength of the humoral immune response was not as great as with infectious BLV [32]. In a previous study it was shown that human antibodies to BLV p24 protein reacted equally well with heat inactivated (boiled) versus unheated purified p24 antigen [16]. Most humans in the USA drink pasteurized dairy products and cooked beef products, which may stimulate anti-BLV antibody production. Therefore, the presence of human antibodies to BLV may be a less accurate indication of BLV infection than the presence of BLV DNA in human cells.

The general assumption about BLV infection of humans is that it is a zoonotic infection, although the possibility of human to human transmission, presumably through blood and/or breast milk, has not been investigated. Epidemiologic observations certainly support a zoonosis. It has been noted for decades that the countries with the highest consumption of dairy products have the highest incidence of breast cancer [33, 34]. Red meat consumption has also been associated with breast cancer incidence [35].

Phylogenetic analyses are usually useful to analyze homology among DNA nucleic acid sequences and determine reservoirs of infectious agents that cause human disease. However, for deltaretroviruses (BLV and HTLV), this type of analysis is extremely difficult because the low mutation rate decreases the number of DNA sequence variations to compare. In the human sequences we report here, there were only occasional single base differences compared to GenBank BLV sequences from cattle specimens (≤3.5% of the LTR region), suggesting close homology with bovine BLV sequences. Other genome regions were often deleted, eliminating any possibility of sequencing them. Phylogenetic trees for different genome regions of BLV isolated from cattle globally have been established [1, 20, 25] and show relatively few variations (base substitutions) among the different geographic areas. What is needed to be conclusive about the homology between human and bovine BLV isolates is more sequences of human isolates for comparison, especially from globally diverse areas. This may take many years to accumulate, especially since multiple BLV genome areas are frequently deleted and therefore whole genome sequencing is not very efficient. This finding of BLV in human blood leukocytes is hopefully, a first step that will inspire other investigators to sample humans in their own global areas, so that eventually collaborative global sequence comparisons among human and bovine sequences could be made and possibly contribute to establishing the reservoir for BLV infection of humans.

## Conclusions

This initial finding of BLV in human blood cells adds a new member to the human tissue types previously found to harbor BLV viz. breast and lung. This is relevant to human cancer because the BLV Tax protein inhibits base excision repair of the oxidative damage to cellular DNA [36], which occurs naturally as a byproduct of normal cell metabolism. This could explain the multiple somatic cell mutations observed in advanced leukemia/lymphomas caused by BLV in cattle [37] and may also explain why human breast and other cancer types have an array of somatic cell mutations [38] that are now being targeted by therapeutic drugs. BLV infection and resultant microRNA production have also been shown to have detrimental effects on the immune system of cattle [39], which might play an important role in advancing the progression of early carcinomas. Thus, BLV has the potential to be an important initiator of cancer in human tissues, and the data reported here further strengthen the evidence that BLV infected cattle pose a likely risk to humans.

### Ackowledgements

We thank the following at Kaiser Permanente Hospital, San Rafael, CA: Karen Kidd, RN for help with specimen acquisition and medical records, and clinical laboratory personnel for drawing blood from study subjects. We are grateful to the following at University of California, Berkeley: Dr. Sangwei Lu for the use of her ELISA reader and Yvonne Hao for assistance with manuscript preparation.

## Abbreviations

BLAST: Basic local alignment search tool
BLV: Bovine leukemia virus
bp: Base pair
DPBS: Dulbecco’s modified phosphate buffered saline
*env*: bovine leukemia virus genome region that codes for the envelope glycoprotein 51
FLK: Fetal lamb kidney
*gag*: region of the bovine leukemia virus genome that codes for the capsid antigen
GAPDH: Glyceraldehyde-3-dehydrogenase
HTLV: Human T-cell leukemia virus
KPM: Kaiser Permanente
KPNC: Kaiser Permanente of Northern California
LTR: Long terminal repeat promoter region of bovine leukemia virus
OR: Odds ratio
*tax*: region of the bovine leukemia virus genome that codes for the transactivating oncogenic protein
UCB: University of California, Berkeley
